# A ‘compare and contrast’ exercise: wrapping versus personalised external aortic root support (PEARS)

**DOI:** 10.1186/s13019-016-0499-7

**Published:** 2016-07-12

**Authors:** Tom Treasure

**Affiliations:** Clinical Operational Research Unit, University College London, London, UK

## Abstract

Wrapping of the aorta and personalised external aortic root support (PEARS) both have the purpose of preventing further expansion of the ascending aorta in order to reduce the risk of aortic dissection and to spare the patient the disastrous consequences of aortic rupture. For the first time, Plonek and colleagues have reported systematically the CT appearances of a series of cases of wrapping. They illustrate the important finding that there are residual spaces between the aorta and the wrap. PEARS by contrast is intimately in contact with the aorta due to its personalised design and is fully incorporated due it construction from a porous mesh. A limitation of PEARS is that it is, of its nature, a planned and elective operation while wrapping can be undertaken during an emergency operation and can be used without prior planning as an intraoperative decision.

## Text

Tomasz Plonek and colleagues [1] provide in their latest article a systematic examination of the CT appearances after the ascending aorta has been wrapped with a vascular prosthesis. This is of considerable interest and relevance. It is a valuable addition to their already published work which includes a systematic review of ‘wrapping’ [2]. They have already reported their version of this technique in operations to restore competence of the aortic valve in ascending aortic aneurysm in two patients [3]. They have also published an elegant biomechanical study of the method [4].

To put this work in context for readers let us first consider the nomenclature. The use of material around the aorta has been referred to as ‘external grafting’ [5], ‘wrapping’ [2], and ‘girdling’ [6]. The external support has been called a ‘jacket’ [7] ‘sleeve’ [8] and a ‘corset’ [3]. In other reports, although it is an intrinsic part of the procedure, the nature of any external support does not appear in the title [9, 10]. The lack of a consistent taxonomy makes reliable searching of the literature difficult. What is described as ‘wrapping’ in Plonek’s paper is the most common terminology. It refers to the use of an off-the-shelf corrugated vascular tube graft, opened along its long axis and wrapped around the ascending aorta and sutured closed as shown in their paper. This is in essence the operation described by Robicsek as a means of reducing the risk of aortic dissection [11]. It is what usually comes to mind when the term ‘wrapping’ is used in meetings or the cardiac surgical literature. It is the reason we carefully avoid the word when writing about personalised external aortic root support (PEARS).

Plonek’s radiological study illustrates well the major short comings of using low porosity relatively rigid graft material for wrapping the aorta: it does not conform well to the aorta and allows accumulation of fluid between the aorta and the support. A pliant, porous mesh [12] on the other hand becomes incorporated in the aorta as has been demonstrated in survival experiments in sheep [13] and at autopsy [14, 15] and reoperation [6]. This avoids the risks of migration and impingement on other structures [15]. The concern about mobility of a vascular graft used as a wrap is heightened by Plonek’s report which shows the persistence of spaces between the aorta and stiff supporting material. The vascular graft is not reliably adherent and so the wrap is routinely stitched to the aorta.

The tube graft covers the aorta from the sino-tubular junction to the brachiocephalic artery. The critical area for aortic dissection is usually in the aortic sinuses which are left unsupported but this limitation is a reasonable exercise of caution with their material. Fashioning the rigid low porosity vascular graft to the sinus would be challenging; positioning it proximal to the coronary arteries to tether it to the aorto-ventricular junction would be high risk. However these are routine steps in the PEARS operation. On the other hand a limitation of PEARS is that it requires a strict imaging protocol, computer aided design, 3-D printing and the manufacture of a customised device so would not be available for an emergency operation. The PEARS approach is not for unplanned use, an unexpected contingency or an intra operative change of plan.

An important element of Plonek’s report is the size reduction achieved by selection of grafts sized to the targeted final diameter of the aorta. In one patient where the diameter of the aorta was reduced to little more than a half of its former size, there was some plication. It appears to be trivial on the CT images. The authors’ explanation is that undersizing is well tolerated because on restoration of the arterial pressure the aorta is evenly expanded to the limits of the vascular graft. In PEARS we have been much more cautious. We have routinely made two supports, one of them with a 5 % reduction in overall diameter over the entire length of the support. This modest size reduction has been used to correct mild degrees of aortic regurgitation. This new evidence from Plonek will make us more confident in reducing the aortic size as part of a PEARS operation.
